# The Manitoba Joint Replacement Registry: validation of a provincial hip and knee arthroplasty registry

**DOI:** 10.2340/17453674.2026.45997

**Published:** 2026-07-01

**Authors:** Colton POITRAS, Christiaan H RIGHOLT, Eric R BOHM

**Affiliations:** 1 Orthopaedic Innovation Centre, Winnipeg, MB; 2 Department of Surgery, University of Manitoba, Winnipeg, MB; 3 College of Pharmacy, University of Manitoba, Winnipeg, MB; 4 Biomedical Engineering Program, University of Manitoba, Winnipeg, MB; 5 Concordia Joint Replacement Group, Winnipeg, MB, Canada

## Abstract

**Background and purpose:**

Regional registries can offer increased dataset linkages and analysis opportunities compared with national registries. However, registry datasets should be properly assessed before their use. The purpose of our study was to validate the completeness and accuracy of the Manitoba Joint Replacement Registry (MJRR) by comparing it with comprehensive databases.

**Methods:**

We identified hip and knee arthroplasty patients in the MJRR ≥ 18 years old who had surgery between 2008 and 2023. We compared our data cohort with the hospital Discharge Abstracts Database (DAD) and the Medical Services Database (MSD) through percentage agreement (proportion identical) and Cohen’s kappa to analyze accuracy and completeness.

**Results:**

We identified 64,472 potential patients included in at least 1 of these 3 datasets. MJRR coverage increased from 68.2% in 2008 to 97.1% in 2023. The percentage agreement between the MJRR, DAD, and MSD was high. No 2 variables matched lower than 90.6%, and all Cohen’s kappa scores were at least within the substantial agreement range (0.61–0.80) with most in the near perfect agreement range (0.81–1.00). Large urban hospitals achieved almost 100% MJRR coverage for all diagnoses, while rural hospitals captured nearly all elective procedures but almost no hip arthroplasty for fractures.

**Conclusion:**

We showed that our regional joint replacement registry contains high completeness of data. We believe that the MJRR is a valuable resource, and its data can be used and trusted for quality initiatives and research projects.

The Manitoba Joint Replacement Registry (MJRR) is a regional hip, knee, and shoulder arthroplasty registry in the Canadian province of Manitoba (population ~1.5 million; 2023 primary arthroplasty incidence per 100,000 person-years: knee, 351; elective hip, 194; hip fracture, 59). While we have assumed the data it contains to be accurate, a formal validation of the MJRR has not previously been done. We aimed to validate the hip and knee information in the MJRR against the arthroplasty information contained in the Manitoba Centre for Health Policy (MCHP) data holdings, a provincial repository housing a wide variety of health data, including hospital information (Discharge Abstracts Database; DAD) and physician billing (Medical Claims Database; MSD).

## Methods

We conducted a regional registry validation study using several databases housed and maintained by the MCHP and Manitoba Health. We followed STROBE reporting guidelines.

### Manitoba Joint Replacement Registry (MJRR)

The MJRR, launched in 2004 as the Winnipeg Regional Health Authority joint replacement registry [1], is a joint replacement registry for hip, knee, and shoulder arthroplasty originally limited to Winnipeg (Manitoba’s main urban center whose metropolitan area houses two-thirds of the provincial population) and now covering all of Manitoba. It receives and stores identical patient demographic and device information to that collected in the Canadian Joint Replacement Registry (CJRR). Additionally, it collects patient-reported outcome measures (PROMs), preoperative self-reported medical and musculoskeletal history, revision status, and self-reported surgical complications occurring within the first postoperative year. Preoperative PROMs collection occurs in the pre-admission clinic, typically 2–4 weeks preoperatively, while the postoperative PROMs are collected by mail at 1 year.

For simplicity, the data collection forms used for the MJRR mirror the forms used in the Canadian Joint Replacement Registry (CJRR). Many distinct versions of the forms have existed since its inception. While all versions collected a core set of patient demographics, procedure type, reason for surgery, and implant characteristics, there were some differences. From 2004/2005–2012/2013, the original CJRR form was in use. In addition to the core set of information already described, it collected information on the patient’s height and weight, previous operations, surgical approach and special techniques, antibiotic use, deep venous thrombosis (DVT) prophylaxis, and operation room (OR) environment details (laminar air flow, exhaust suits). Unfortunately, data capture with this form remained below 50% nationally, and in 2012/13 this form was updated to the minimum dataset form (MDS). This form removed the additional “non-core” data elements collected in the original form and updated the diagnosis and reasons for revision options through an international survey of existing registries. In 2013/14, a second version of the MDS was launched. This form, still in use today, added additional options to existing fields.

The MJRR currently collects preoperative and 1-year postoperative Oxford Knee Score [2], Oxford Hip Score [3], European Quality of Life Index (EQ-5D) score [4], and postoperative 1- and 2-year patient satisfaction on a 5-point Likert scale [5]. The 12-item Short Form Survey (SF-12) [6] was collected between 2004 and 2020 before being changed to the EQ-5D to align with national recommendations [7]. The MJRR also collects preoperative pain/functional limitations and medical history. Preoperative PROM information is collected in the pre-admission clinic by a clinic nurse [1]. Postoperative data is collected through mail-out, and is handled and input by MJRR registry staff [1]. The inclusion of such PROMs in regional registries has resulted in improved outcome reports for patients [1,5,8,9].

### Other data sources

The Population Registry tracks addresses, dates of birth, death, and health insurance coverage for all insured persons. Since 1971, the hospital Discharge Abstracts Database (DAD) has recorded virtually all inpatient services provided by hospitals in the province [10]. The data collected comprises demographic as well as diagnosis and treatment information including primary diagnosis and service or procedure codes, coded using the International Classification of Diseases, Ninth Revision, Clinical Modification (ICD-9-CM) before April, 2004, and the ICD-10-CA (Canadian adaptation of the ICD-10) and the Canadian Classification of Health Interventions (CCI) thereafter. The DAD has also collected similar information for outpatients since 2004. The Medical Services Database (MSD), also in operation since 1971, collects similar information, based on physician fee-for-service or shadow billing, on services provided by physicians in offices, hospitals, and outpatient departments across the province [10].

### Study cohort

Our source population consisted of all persons 18 years of age or older who were insured through Manitoba Health and registered in the Manitoba Health Insurance Registry (MHIR) in the study period between January 1, 2008 (the beginning of our MJRR information) and December 31, 2023 (the most recent available data). Our arthroplasty cohorts, one each for the MJRR, DAD, and MSD, consist of all members of the source population, in each respective set, who received knee or hip arthroplasty in Manitoba while insured through provincial health coverage (virtually all legal residents) during the study period (see Supplementary Table 1 for details). We excluded any patient who did not have a valid Manitoba health insurance number (PHIN), those who received surgery out of province, or those without a valid surgery or hospital admission date.

### Data linkage

The MJRR, DAD, and MSD were linked through a unique key consisting of a unique patient identifier, surgery date, joint (hip or knee), side (left or right), and, when available, type (primary or revision). Any additional surgery with duplicate combination of these variables was excluded.

### Statistics

We compared different relevant variables such as procedure, hospital, bilateral status, and diagnosis across the 3 sets through percentage agreement (proportion identical) and Cohen’s kappa. Cohen’s kappa measures inter-rater reliability and incorporates the probability of agreement occurring by chance and has been used to validate arthroplasty registry data in a variety of jurisdictions [11-14]. Kappa scores are defined in ranges with 0.61–0.80 viewed as substantial agreement and 0.81–1.00 viewed as near perfect agreement [15]. The percentage agreement across sets was measured through exact matching; a success was an exact match, and a failure was a non-match. We assessed the coverage of surgeries that existed in the MJRR against those that existed outside the MJRR over time separately for joint, hospital, and sex to understand the differences between reporting rates over time for each study variable. Kappa scores and percentage agreement were compared between the MJRR, DAD, and MSD.

We used Stata 16 MP (StataCorp, College Station, TX, USA) for all data analysis. We used SAS Enterprise Guide 8.3 (SAS Institute, Cary, NC, USA) for data cleaning and linkage.

### Ethics, data sharing plan, funding, use of AI, and disclosures

This study was approved by the University of Manitoba Research Ethics Board and by the Provincial Health Research Privacy Committee. Data used in this article was derived from administrative health and social data as a secondary use. The data was provided under specific data-sharing agreements only for approved use at the MCHP. The original source data is not owned by the researchers or MCHP and as such cannot be provided to a public repository. The original data source and approval for use has been noted in the acknowledgments of the article. Where necessary, source data specific to this article or project may be reviewed at MCHP with the consent of the original data providers, along with the required privacy and ethical review bodies. This study was funded by the Concordia Foundation, the University of Manitoba’s Alexander Gibson Research Fund, and the University of Manitoba’s Department of Surgery Annual GFT Research Grant. The opinions presented in the report do not necessarily reflect those of the funders. No AI was used in the data collection, data preparation, data analysis, interpretation of the results, creation of figures or tables, drafting of the manuscript, or copy-editing of the manuscript. We used Microsoft Word (Microsoft Corp, Redmond, WA, USA) for manuscript preparation, which may have included AI in some of its proofing tools.

CP and CHR are employed by the Orthopaedic Innovation Centre. CHR has received institutional research funding from Pfizer for an unrelated study. CHR and ERB have received institutional research funding from DePuy-Synthes, Zimmer-Biomet, Smith & Nephew, Stryker, and Hip Innovation Technology for unrelated studies. ERB has received consulting fees from Stryker. Complete disclosure of interest forms according to ICMJE are available on the article page, doi: 10.2340/17453674.2026.45997

## Results

**Figure 1:**
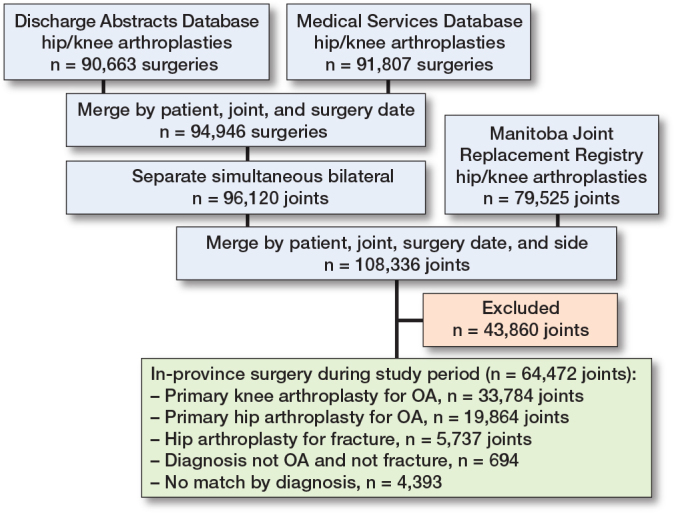
Cohort assembly and linkage of registry datasets: patient flowchart.

Our study cohort consisted of 64,472 patients, who had 33,784 primary knee arthroplasties for osteoarthritis (mostly total knee arthroplasty), 19,864 primary hip arthroplasties (mostly total hip arthroplasty), 5,737 arthroplasties to treat hip fractures (Figure 1; Table 1), 694 for other non-fracture non-OA diagnoses, and 4,393 that did not match by diagnosis (not shown in Table 1).

**Table 1:** Number (%) of primary hip and knee arthroplasties according to patient and surgical characteristics by type of surgery and primary diagnosis among patients included in all 3 data sources

Item	Knee OA	Hip OA	Hip fracture
	n = 33,784	n = 19,864	n = 5,737
Sex			
Male	13,491 (40)	9,248 (47)	1,839 (32)
Female	20,293 (60)	10,616 (53)	3,898 (68)
Procedure			
TKA	30,238 (90)	0 (0.0)	0 (0.0)
UKA	1,817 (5.4)	0 (0.0)	0 (0.0)
THA	0 (0.0)	19,838 (100)	1,112 (19)
Hemiarthroplasty	0 (0.0)	11 (0.1)	4,457 (78)
Differs by data source	1,729 (5.1)	15 (0.1)	168 (2.9)
Simultaneous bilateral			
Bilateral	1,178 (3.5)	275 (1.4)	7 (0.1)
Unilateral	32,042 (95)	19,364 (98)	5,675 (99)
Differs by data source	564 (1.7)	225 (1.1)	55 (1.0)
Hospital			
I	11,713 (35)	7,875 (40)	1,348 (24)
II	13,299 (39)	7,276 (37)	1,299 (23)
III	3,191 (9.4)	2,325 (12)	344 (6.0)
IV	3,874 (12)	1,488 (7.5)	680 (12)
V	73 (0.2)	97 (0.5)	672 (12)
VI	1,379 (4.1)	658 (3.3)	1,334 (23)
VII	45 (0.1)	S	S
Differs by data source	210 (0.6)	S	S

OA = osteoarthritis; TKA = total knee arthroplasty; UKA = unicompartmental knee arthroplasty; THA = total hip arthroplasty; I = teaching hospital; II = high-volume urban hospital; III, IV = rural hospital; V, VI, VII = low-volume urban hospital. S indicates the cell’s value is suppressed to avoid deriving cells with values < 6.

### Completeness

**Figure 2:**
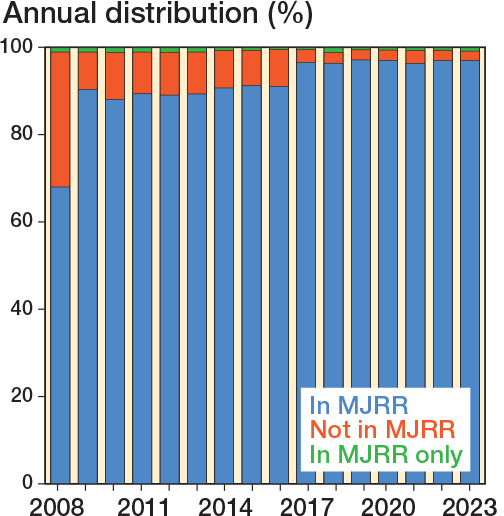
Proportion of hip and knee arthroplasty according to Manitoba Joint Replacement Registry (MJRR) status by year.

**Figure 3:**
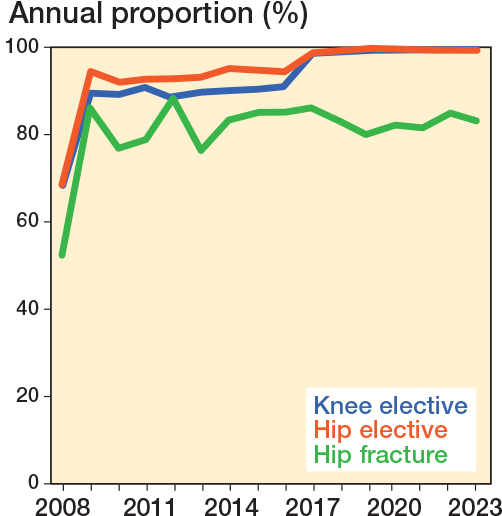
Proportion of hip and knee arthroplasty according to joint by year.

Overall, MJRR coverage increased from 68% in 2008 to 97% in 2023 (Figure 2; Table S2). Completeness in 2023 was 99.5% for elective knees, 99.5% for elective hips, and 83.3% for hip fractures. (Figure 3; Table S3). Large changes in MJRR coverage occurred from 2008 (68%) to 2009 (91%) and 2016 (91%) to 2017 (97%). There were no major differences in coverage by sex (Figure S1; Table S4). There was high agreement (> 96%) across all 3 data sources in primary vs revision status; kappa was > 0.8.

**Figure 4:**
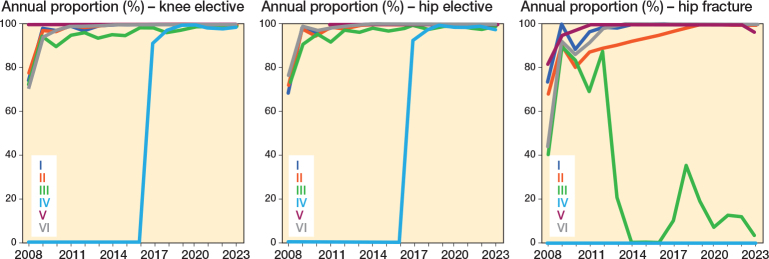
Proportion of hip and knee arthroplasty according to hospital by year. I = teaching hospital; II = high-volume urban hospital; III, IV = rural hospital; and V, VI = low-volume urban hospital.

Non-Winnipeg hospitals began reporting to the CJRR later than Winnipeg hospitals, and their coverage has still not caught up to Winnipeg hospitals. The 2 non-Winnipeg hospitals report almost all elective procedures and almost no hip arthroplasty for fractures (Figure 4; Table S5). Winnipeg hospitals reported high coverage, with little difference between elective and non-elective procedures. The highest-volume arthroplasty units post elective coverages of 100% and have had near-perfect coverage since 2014 (Figure 4; Table S5).

### Agreement

The percentage agreement between the MJRR, the DAD, and the MSD was high. Percentage agreement for all variables was higher than 90% and 9 of 13 were above 95% (Table 2). Diagnosis and procedure agreement across the 3 sets have the least agreement, while hospital, surgery, and bilateral status have the highest. All kappa scores when comparing the MJRR against the DAD are in the “near perfect agreement” range (0.81–1.00); all kappa scores for any variable from any set to set comparison are in at least the “substantial agreement” range (0.61–0.80), and all scores that fall in the “substantial” range are near its upper bound (Table 2).

**Table 2:** Agreement (%) and Cohen’s kappa (κ) of patient and surgical characteristics between the MJRR, DAD, and MSD

Item	Agreement (%)	Kappa (κ)
MJRR vs DAD		
Hospital	99.5	0.99
Simultaneous bilateral	99.8	0.96
Primary vs revision	98.9	0.93
Diagnosis	97.2	0.92
Procedure	95.8	0.93
MJRR vs MSD		
Hospital	N/A **^a^**	N/A **^a^**
Simultaneous bilateral	98.7	0.79
Primary vs revision	97.2	0.82
Diagnosis	92.9	0.77
Procedure	90.6	0.85
DAD vs MSD		
Hospital	N/A **^a^**	N/A **^a^**
Simultaneous bilateral	98.6	0.75
Primary vs revision	96.7	0.80
Diagnosis	93.3	0.80
Procedure	90.8	0.85

a MSD = Medical Services Database does not include hospital.

N/A = not applicable; MJRR = Manitoba Joint Replacement Registry; DAD = Hospital Discharge Abstracts Database; MSD = Medical Services Database.

### Diagnoses

Within the main indications (osteoarthritis and hip fractures), percentage agreement for procedure was above 91% for every comparison, and among every variable above 97% for 17 of 21 (Table 3). The kappa scores reflect this high agreement with nearly all scores falling in the “almost perfect” to “substantial” ranges. Some kappa scores are low, such as among procedure for hip osteoarthritis reported in the “poor’ range (15) (k = 0.19, 0.00, 0.04; Table 3), and among simultaneous bilateral for “hip fracture” (k = 0.14, 0.13; Table 3). Overall, the MJRR and DAD have higher agreement and kappa scores between them than they share with the MSD. There were widespread increases in agreement and kappa scores from 2008 to 2023 (Table S6). The year-to-year outcomes among any category increased, generally, monotonically. All agreements in 2023 were above 96%, and all fall within the “near perfect” kappa range. > 95% agreement and “near-perfect” kappa scores for all variables and all sets have been near universally true since 2017 (Table S6).

**Table 3:** Agreement (%) and Cohen’s kappa (κ) of patient and surgical characteristics between the MJRR, DAD, and MSD when matching procedures on joint, diagnosis, and revision status

Item	Knee	Hip	Hip
	OA	OA	fracture
Agreement (%)			
Hospital: MJRR vs DAD	99.6	99.6	99.4
Procedure: MJRR vs DAD	96.0	99.5	91.8
Procedure: MJRR vs MSD	97.9	99.8	92.1
Procedure: DAD vs MSD	97.2	99.6	93.1
Simultaneous bilateral: MJRR vs DAD	99.6	99.9	99.9
Simultaneous bilateral: MJRR vs MSD	98.6	98.9	98.8
Simultaneous bilateral: DAD vs MSD	98.4	98.9	98.9
Kappa (κ)			
Hospital: MJRR vs DAD	0.99	0.99	0.99
Procedure: MJRR vs DAD	0.57	0.18	0.78
Procedure: MJRR vs MSD	0.75	0.00	0.79
Procedure: DAD vs MSD	0.75	0.04	0.80
Simultaneous bilateral: MJRR vs DAD	0.95	0.98	0.75
Simultaneous bilateral: MJRR vs MSD	0.84	0.72	0.14
Simultaneous bilateral: DAD vs MSD	0.80	0.70	0.13

For abbreviations, see Table 2.

## Discussion

We aimed to validate the completeness and accuracy of the Manitoba Joint Replacement Registry (MJRR) by comparing it with other comprehensive databases. We found high completeness of the MJRR across any hip or knee arthroplasty when compared with the DAD and MSD at 97.1% in 2023. This is beyond the generally accepted standard of compliance (90-95%) [16-18]. In comparison, national registries report similar completeness, such as in the Norwegian Arthroplasty Register (NAR) (96.6% for primary knee replacement; 97.0% for primary hip) [19], 96% for primary THA in the Finnish Arthroplasty Registry (FAR) [20], but also may do worse, such as a 73.9% completeness for all hip and knee arthroplasty in the CJRR [21]. The MJRR was found to be 99.5% complete for elective hips in 2023, which is higher than the completeness reported in the NAR and FAR under the assumption that most elective hips are being treated with THA in all jurisdictions, and 99.5% complete for elective knees, also higher than the NAR. The large changes in MJRR coverage from 2008 to 2009 reflect the staged hospital-by-hospital rollout of the registry, and from 2016 to 2017 when reporting became mandatory for funding brought the last hospital on board (Figure 4; Table S5). We found high agreement and consistent “near perfect” kappa scores between the MJRR, DAD. and MSD for the listed variables (Tables 2 and 3), which is well within the standard of compliance. Other regional registries have been shown to be comparable to national data holdings, such as the regional Tyrolean hip arthroplasty registry in Austria reporting a capture rate of 98% of all primary THAs [22] and the Emilia-Romagna arthroplasty registry in Italy reporting a 96% capture rate of all hip, knee, and shoulder operations in 2021 [23].

Whenever 2 datasets are mostly the same, e.g., almost all (> 99.9%) hip osteoarthritis was treated with total hip arthroplasty, even a single difference among the remaining 0.01% causes the kappa score to overinflate its interpretation of randomness. For such counts, it is important to consider both percentage agreement and kappa together. This occurred for outcomes such as simultaneous bilateral hip fracture (k = 0.14, 0.13; see Table 3), or procedure details for hip osteoarthritis (k = 0.18, 0.00, 0.04; Table 3), where percentage agreement was ≥ 98.8% and ≥ 99.5%, respectively.

We found that hip fracture arthroplasty is not captured as well in the MJRR, particularly recently and among non-Winnipeg hospitals. Whether the non-elective/acute nature of hip fractures causes a differing workflow, submission protocols are misunderstood for these cases, or something else, this is an area where the MJRR lacks information. While the Danish Hip Arthroplasty reported that THA coverage was 94% from 1995–2000, with a lower predictive value for fracture (30%) than osteoarthritis (85%) [24], to our knowledge, this is the first arthroplasty registry validation study that examined the differences in capture between elective and emergent procedures rather than completeness. This problem may be unique to the MJRR or may explain lower than expected capture among other regional or national registries.

The data itself provides no guidance on what should be taken as authoritative when there are differences. DAD data entry is performed by trained hospital staff specialized in coded hospital abstracts; MJRR data is entered by surgeons and their surgical staff right from the OR. In our opinion, the person completing the operation would be the most reliable source as no interpretation is required; that is not to say that the trained hospital staff are unreliable, rather the opposite, as the agreement levels found between the MJRR and the DAD validate both as reliable data sources for hip and knee arthroplasty information. The MJRR has the added benefit of being specific to arthroplasty while including self-reported medical history and PROMs.

### Strengths

Manitoba’s main urban center, Winnipeg and its metropolitan area, contains two-thirds of the province’s population and is the site of Manitoba’s only tertiary hospitals, which suggests an environment for high completeness. Another major strength of this study is its internal validity. It is unlikely that anything other than the high quality of all datasets drives the high agreement rates between the MJRR, DAD, and MSD. The information is from distinct sources that match due to consistency across datasets due to high-quality data entry and storage.

### Limitations

We tested the agreement of only a finite number of variables, which may not be generalizable to the quality of other variables. Another limitation is that we matched on the joint and arthroplasty side, so misclassification in either will cause a non-match across sets, but the number of possible non-matches is low (n = 298 based on person and surgery date); these non-matches would further contribute to the completeness of the MJRR, which already reported 97% in 2023 and are non-consequential. Another limitation is that this study is only relevant to the MJRR against the best (known) sources of completeness regarding arthroplasty data in Manitoba. This may not be transferable to other regional joint replacement registries, as Manitoba historically has had very high submission rates, was the first province with mandatory electronic submission, and has 1 major city (Winnipeg) that contains all its tertiary hospitals.

### Conclusion

This study showed completeness and accuracy of the MJRR for hip and knee arthroplasty in Manitoba. This shows that regional joint registries can deliver high-quality information. However, coverage of hip fractures outside of Winnipeg is lacking. The MJRR is a useful resource, and its data should be used for quality research.

### Supplementary data

Figure S1 and Table S1–S6 are available as supplementary data on the article homepage, doi: 10.2340/17453674.2026.45997

## Supplementary Material



## Notes

CP: methodology, software, formal analysis, data curation, writing—original draft, visualization. CHR: conceptualization, methodology, software, formal analysis, resources, writing—review and editing, supervision, project administration, acquisition. ERB: conceptualization, resources, writing—review and editing, supervision, funding acquisition.

The authors acknowledge the Manitoba Centre for Health Policy for use of data contained in the Manitoba Population Research Data Repository under project # 2024-035 (PHRPC # P2024-24, REB # HS26434(H2024:131)). The results and conclusions are those of the authors and no official endorsement by the Manitoba Centre for Health Policy, the Winnipeg Regional Health Authority, Shared Health, Manitoba Health, or other data providers is intended or should be inferred. Data used in this study is from the Manitoba Population Research Data Repository housed at the Manitoba Centre for Health Policy, University of Manitoba and was derived from data provided by Manitoba Health.

Handling co-editors: Marianne Westberg and Philippe Wagner

*Acta* thanks Torbjørn Berge Kristensen and other anonymous reviewers for help with peer review of this manuscript.
