# Supplementary material for: The Manitoba Joint Replacement Registry: validation of a provincial hip and knee arthroplasty registry
Source: Acta Orthop. 2026 Jul 1;97:442–7. doi: 10.2340/17453674.2026.45997 (PMC13320592; doi:10.2340/17453674.2026.45997)
Supplement: Supplementary file 1 [file ActaO-97-45997-s1.pdf]

Supplementary material to "**The Manitoba Joint Replacement Registry: Validation of a provincial hip and knee arthroplasty registry**" by Colton Poitras, Christiaan H. Righolt, and Eric R. Bohm

Supplementary figures

Figure S1. Proportion of hip and knee arthroplasty according to sex and elective status by year

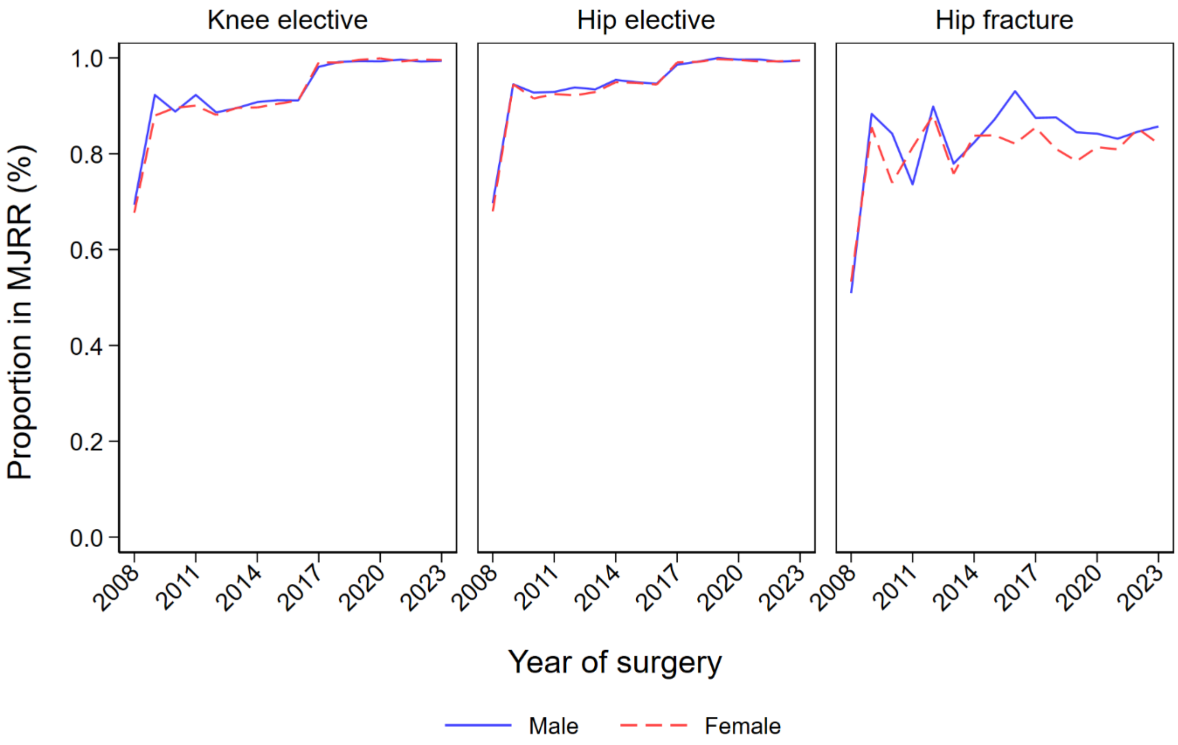

Supplementary tables

Table S1. List of hip and knee procedure classifications by ICD-10-CA and ICD-9-CM intervention codes and historical and current Manitoba physician tariff codes.

Coding system	Joint	Intervention code	Intervention procedure
ICD-10-CA	Hip	1.SQ.53.LA[1]	Acetabular arthroplasty
ICD-10-CA	Hip	1.VA.53.LA[1]	Hemiarthroplasty/THA
ICD-10-CA	Hip	1.VA.53.LL[1]	Hemiarthroplasty/THA
ICD-9-CM	Hip	0070	Cup and stem revision
ICD-9-CM	Hip	0071	Cup revision
ICD-9-CM	Hip	0072	Stem revision
ICD-9-CM	Hip	0073	Head and/or liner exchange
ICD-9-CM	Hip	0085	Hip resurfacing
ICD-9-CM	Hip	0086	Hip resurfacing
ICD-9-CM	Hip	0087	Hip resurfacing
ICD-9-CM	Hip	8151	THA
ICD-9-CM	Hip	8152	Hemiarthroplasty
ICD-9-CM	Hip	8153	Unknown revision
Tariff[2]	Hip	1414	THA
Tariff[2]	Hip	1415	THA
Tariff[2]	Hip	1416	THA
Tariff[2]	Hip	1417	Liner exchange
Tariff[2]	Hip	1418	Head exchange
Tariff[2]	Hip	1419	Head and liner exchange
Tariff[2]	Hip	1420	Cup or stem revision
Tariff[2]	Hip	1421	Two component revision
Tariff[2]	Hip	1423	Hemiarthroplasty
Tariff[2]	Hip	1424	Hemiarthroplasty
ICD-10-CA	Knee	1.VG.53.LA-PM[3]	Single component arthroplasty
ICD-10-CA	Knee	1.VG.53.LA-PN[3]	Dual component arthroplasty
ICD-10-CA	Knee	1.VG.53.LA-PP[3]	Tri component arthroplasty
ICD-10-CA	Knee	1.VG.53.LA-PR	Liner exchange
ICD-10-CA	Knee	1.VG.53.LA-SL	Cement spacer
ICD-10-CA	Knee	1.VP.53.LA-PM	Patella resurfacing only
ICD-9-CM	Knee	0080	Femoral, tibial and patellar revision
ICD-9-CM	Knee	0081	Tibial component replacement
ICD-9-CM	Knee	0082	Femoral component replacement
ICD-9-CM	Knee	0083	Patella resurfacing only
ICD-9-CM	Knee	0084	Insert exchange
ICD-9-CM	Knee	8154	Knee arthroplasty
ICD-9-CM	Knee	8155	Unknown revision
Tariff[2]	Knee	1402	TKA with patella resurfacing
Tariff[2]	Knee	1403	TKA without patella resurfacing
Tariff[2]	Knee	1404	UKA
Tariff[2]	Knee	1405	Patella resurfacing only
Tariff[2]	Knee	1407	TKA
Tariff[2]	Knee	1408	Insert exchange
Tariff[2]	Knee	1409	Two component revision

[1] Specific subcodes (PM, PN, PR, SL) attached to 1.SQ.53,1.VA.53 provide further details on the procedure; [2] Current physician tariffs since 2006; [3] Extension codes denote which specific compartment(s) are replaced

Table S2. Number of hip and knee arthroplasties according to Manitoba Joint Replacement Registry (MJRR) status by year. Values are count (%)

	**2008 n = 3,207**	**2009 n = 2,980**	**2010 n = 3,371**	**2011 n = 3,525**	**2012 n = 3,468**	**2013 n = 3,623**	**2014 n = 3,638**	**2015 n = 3,751**	**2016 n = 3,875**	**2017 n = 4,064**	**2018 n = 4,463**	**2019 n = 5,022**	**2020 n = 4,195**	**2021 n = 4,045**	**2022 n = 5,154**	**2023 N = 6,254**
Dataset coverage																
In the MJRR	2,185 (68.2)	2,690 (90.6)	2,923 (88.3)	3,106 (89.5)	3,030 (89.2)	3,223 (89.5)	3,306 (90.9)	3,425 (91.4)	3,532 (91.2)	3,927 (96.7)	4,305 (96.5)	4,884 (97.3)	4,070 (97.0)	3,897 (96.4)	5,007 (97.1)	5,980 (97.1)
Not in the MJRR	992 (30.9)	250 (8.4)	350 (10.6)	329 (9.5)	330 (9.7)	348 (9.7)	308 (8.5)	302 (8.1)	327 (8.4)	118 (2.9)	110 (2.5)	112 (2.2)	107 (2.6)	121 (3.0)	118 (2.3)	131 (2.1)
In the MJRR only	29 (0.9)	29 (1.0)	38 (1.1)	35 (1.0)	35 (1.0)	29 (0.8)	22 (0.6)	20 (0.5)	13 (0.3)	17 (0.4)	47 (1.1)	23 (0.5)	17 (0.4)	25 (0.6)	29 (0.6)	46 (0.7)

Table S3. Proportion of hip and knee arthroplasty in MJRR according to elective joint by year. Values are count (%)

	Knee elective	Hip elective	Hip fracture
2008	1,077 (68.2)	514 (68.6)	100 (52.6)
2009	1,404 (89.6)	751 (94.5)	177 (86.3)
2010	1,491 (89.2)	899 (92.0)	203 (76.9)
2011	1,696 (90.8)	939 (92.6)	224 (78.9)
2012	1,589 (88.2)	928 (92.9)	236 (88.4)
2013	1,751 (89.5)	989 (93.1)	189 (76.2)
2014	1,668 (90.1)	1,122 (95.2)	265 (83.3)
2015	1,751 (90.7)	1,168 (94.9)	284 (85.0)
2016	1,828 (91.1)	1,174 (94.5)	296 (85.3)
2017	2,119 (98.7)	1,273 (98.8)	336 (86.2)
2018	2,381 (99.1)	1,371 (99.2)	322 (83.2)
2019	2,663 (99.5)	1,640 (99.9)	324 (80.2)
2020	2,148 (99.7)	1,259 (99.6)	390 (82.3)
2021	1,930 (99.4)	1,349 (99.5)	403 (81.7)
2022	2,625 (99.5)	1,691 (99.3)	465 (85.0)
2023	3,414 (99.5)	1,890 (99.5)	444 (83.3)

Table S4. Proportion of hip and knee arthroplasty in MJRR according to sex and elective status by year. Values are count (%)

	Male - knee elective	Female - knee elective	Male - hip elective	Female - hip elective	Male - hip fracture	Female - hip fracture
2008	414 (69.2)	663 (67.5)	245 (69.6)	269 (67.8)	28 (50.9)	72 (53.3)
2009	570 (92.2)	834 (87.9)	341 (94.5)	410 (94.5)	53 (88.3)	124 (85.5)
2010	584 (88.8)	907 (89.5)	394 (92.7)	505 (91.5)	64 (84.2)	139 (73.9)
2011	654 (92.2)	1,042 (90.0)	403 (92.9)	536 (92.4)	67 (73.6)	157 (81.3)
2012	589 (88.6)	1,000 (88.0)	425 (93.8)	503 (92.1)	71 (89.9)	165 (87.8)
2013	705 (89.5)	1,046 (89.6)	481 (93.4)	508 (92.9)	52 (77.6)	137 (75.7)
2014	669 (90.8)	999 (89.7)	541 (95.4)	581 (94.9)	84 (82.4)	181 (83.8)
2015	674 (91.2)	1,077 (90.4)	546 (95.0)	622 (94.8)	102 (87.2)	182 (83.9)
2016	752 (91.2)	1,076 (91.1)	512 (94.6)	662 (94.4)	94 (93.1)	202 (82.1)
2017	850 (98.2)	1,269 (99.1)	628 (98.6)	645 (99.1)	112 (87.5)	224 (85.5)
2018	960 (99.2)	1,421 (99.0)	643 (99.2)	728 (99.2)	113 (87.6)	209 (81.0)
2019	1,090 (99.4)	1,573 (99.6)	771 (100.0)	869 (99.8)	98 (84.5)	226 (78.5)
2020	875 (99.3)	1,273 (99.9)	599 (99.7)	660 (99.5)	128 (84.2)	262 (81.4)
2021	824 (99.6)	1,106 (99.3)	636 (99.7)	713 (99.3)	148 (83.1)	255 (81.0)
2022	1,089 (99.3)	1,536 (99.7)	796 (99.3)	895 (99.3)	165 (84.6)	300 (85.2)
2023	1,347 (99.4)	2,067 (99.6)	885 (99.4)	1,005 (99.5)	150 (85.7)	294 (82.1)

Table S5. Proportion of hip and knee arthroplasty in MJRR according to hospital by year. Values are count (%)

	I	II	III elective	III fracture	IV elective	IV fracture	V	VI
2008	864 (73.3)	695 (75.7)	31 (68.9)	S	0 (0.0)	0 (0.0)	27 (84.4)	333 (69.4)
2009	1,030 (98.0)	866 (97.3)	126 (93.3)	17 (89.5)	0 (0.0)	0 (0.0)	45 (95.7)	498 (95.2)
2010	1,155 (96.6)	818 (95.4)	242 (92.0)	26 (83.9)	0 (0.0)	0 (0.0)	42 (95.5)	603 (95.9)
2011	1,179 (98.7)	816 (98.8)	269 (93.7)	29 (69.0)	0 (0.0)	0 (0.0)	47 (100.0)	705 (97.9)
2012	1,208 (97.9)	1,346 (98.5)	265 (96.7)	21 (87.5)	0 (0.0)	0 (0.0)	53 (96.4)	108 (98.2)
2013	1,346 (99.5)	1,450 (99.4)	275 (94.8)	7 (21.2)	0 (0.0)	0 (0.0)	43 (100.0)	88 (100.0)
2014	1,322 (100.0)	1,409 (99.9)	344 (96.6)	0 (0.0)	0 (0.0)	0 (0.0)	57 (100.0)	159 (100.0)
2015	1,328 (100.0)	1,489 (100.0)	358 (96.0)	0 (0.0)	0 (0.0)	0 (0.0)	50 (100.0)	186 (100.0)
2016	1,371 (100.0)	1,507 (100.0)	370 (98.1)	0 (0.0)	0 (0.0)	0 (0.0)	48 (98.0)	220 (100.0)
2017	1,425 (100.0)	1,472 (100.0)	374 (98.9)	S	334 (91.8)	0 (0.0)	50 (100.0)	238 (100.0)
2018	1,669 (100.0)	1,528 (99.9)	406 (96.9)	S	375 (97.4)	0 (0.0)	52 (100.0)	240 (100.0)
2019	1,665 (100.0)	2,140 (100.0)	433 (98.0)	S	514 (99.6)	0 (0.0)	102 (99.0)	6 (100.0)
2020	1,423 (100.0)	1,641 (100.0)	361 (99.2)	S	477 (99.4)	0 (0.0)	148 (98.7)	a
2021	1,339 (100.0)	1,505 (100.0)	342 (98.8)	S	575 (98.6)	0 (0.0)	110 (100.0)	a
2022	1,733 (100.0)	1,976 (100.0)	428 (98.8)	S	725 (98.4)	0 (0.0)	122 (98.4)	a
2023	2,393 (100.0)	2,233 (100.0)	691 (99.1)	S	532 (98.5)	0 (0.0)	101 (98.1)	a

[a] Hip and knee arthroplasty stopped at VI after 2019. I = Teaching hospital; II = High volume urban hospital; III,IV = Rural hospital; V,VI = Low volume urban hospital. S indicates the cell's value is suppressed to avoid deriving cells with values < 6.

Table S6: Agreement (%) and Cohen's kappa (κ) of patient and surgical characteristics between the MJRR, DAD, and MSD by year

	2008	2009	2010	2011	2012	2013	2014	2015	2016	2017	2018	2019	2020	2021	2022	2023
Agreement																
Hospital: MJRR vs. DAD	93.5	97.8	99.7	99.0	99.5	100.0	99.9	100.0	100.0	100.0	100.0	100.0	100.0	100.0	100.0	100.0
Procedure: MJRR vs. DAD	97.0	96.5	95.2	93.7	95.3	98.9	99.0	99.2	99.0	97.8	97.6	96.9	97.2	96.0	95.7	96.1
Procedure: MJRR vs. MSD	98.4	98.1	96.7	96.1	96.0	98.5	99.3	99.1	99.0	98.9	97.8	98.0	98.2	98.4	98.2	98.1
Procedure: DAD vs. MSD	98.4	97.9	97.7	97.2	98.0	98.2	99.0	98.6	98.5	98.3	98.5	97.5	97.6	96.1	96.2	96.6
Simul bilateral: MJRR vs. DAD	99.9	100.0	100.0	99.9	99.9	99.8	100.0	100.0	100.0	100.0	99.7	99.8	99.7	99.4	99.5	99.5
Simul bilateral: MJRR vs. MSD	99.9	99.9	99.9	99.4	99.6	99.8	80.7	99.9	99.9	99.9	99.8	99.8	99.9	99.9	99.8	99.7
Simul bilateral: DAD vs. MSD	99.8	99.9	99.9	99.4	99.6	99.8	80.8	99.9	99.9	99.9	99.6	99.7	99.7	99.4	99.5	99.4
Diagnosis: MJRR vs. DAD	87.6	92.8	91.9	94.7	95.2	96.2	98.1	97.8	98.1	97.9	99.4	99.9	99.7	99.8	99.7	99.8
Diagnosis: MJRR vs. MSD	87.3	89.9	88.2	92.4	92.6	92.0	93.6	94.8	94.9	96.4	95.6	94.7	93.1	95.3	96.1	96.3
Diagnosis: DAD vs. MSD	82.3	90.9	94.2	95.5	94.5	94.1	94.3	95.6	95.1	96.4	95.9	95.4	94.4	95.8	97.0	96.7
Kappa (κ)																
Hospital: MJRR vs. DAD	0.91	0.97	1.00	0.99	0.99	1.00	1.00	1.00	1.00	1.00	1.00	1.00	1.00	1.00	1.00	1.00
Procedure: MJRR vs. DAD	0.94	0.93	0.91	0.88	0.91	0.98	0.98	0.99	0.98	0.96	0.96	0.95	0.95	0.93	0.93	0.93
Procedure: MJRR vs. MSD	0.97	0.96	0.94	0.93	0.93	0.97	0.99	0.98	0.98	0.98	0.96	0.96	0.97	0.97	0.97	0.97
Procedure: DAD vs. MSD	0.97	0.96	0.96	0.95	0.96	0.97	0.98	0.98	0.97	0.97	0.97	0.96	0.96	0.94	0.94	0.94
Simul bilateral: MJRR vs. DAD	0.99	0.99	0.99	0.99	0.98	0.97	0.99	1.00	0.99	0.99	0.95	0.96	0.96	0.91	0.93	0.90
Simul bilateral: MJRR vs. MSD	0.98	0.99	0.99	0.87	0.93	0.95	0.10	0.99	0.98	0.98	0.97	0.95	0.98	0.98	0.97	0.95
Simul bilateral: DAD vs. MSD	0.97	0.97	0.98	0.86	0.92	0.96	0.09	0.99	0.99	0.97	0.94	0.92	0.95	0.91	0.92	0.87
Diagnosis: MJRR vs. DAD	0.49	0.70	0.70	0.73	0.48	0.74	0.92	0.90	0.92	0.92	0.97	0.99	0.99	0.99	0.99	0.99
Diagnosis: MJRR vs. MSD	0.43	0.55	0.54	0.57	0.28	0.51	0.71	0.76	0.76	0.84	0.79	0.74	0.75	0.83	0.84	0.82
Diagnosis: DAD vs. MSD	0.35	0.58	0.72	0.75	0.71	0.67	0.73	0.78	0.76	0.83	0.79	0.76	0.78	0.84	0.86	0.82

*MSD does not include hospital. MJRR = Manitoba Joint Replacement Registry; DAD = Hospital Discharge Abstracts Database; MSD = Medical Services Database; Simul. bilat. = Simultaneous bilateral
